# Hybrid 3D Printing of Advanced Hydrogel-Based Wound Dressings with Tailorable Properties

**DOI:** 10.3390/pharmaceutics13040564

**Published:** 2021-04-16

**Authors:** Marko Milojević, Gregor Harih, Boštjan Vihar, Jernej Vajda, Lidija Gradišnik, Tanja Zidarič, Karin Stana Kleinschek, Uroš Maver, Tina Maver

**Affiliations:** 1 Faculty of Medicine, Institute of Biomedical Sciences, University of Maribor, Taborska Ulica 8, SI-2000 Maribor, Slovenia; marko.milojevic1@um.si (M.M.); bostjan.vihar@um.si (B.V.); jernej.vajda1@um.si (J.V.); lidija.gradisnik@um.si (L.G.); tanja.zidaric@um.si (T.Z.); 2 Department of Pharmacology, Faculty of Medicine, University of Maribor, Taborska Ulica 8, SI-2000 Maribor, Slovenia; 3 Laboratory for Intelligent CAD Systems, Faculty of Mechanical Engineering, University of Maribor, Smetanova 17, SI-2000 Maribor, Slovenia; gregor.harih@um.si; 4 IRNAS Ltd., Valvasorjeva 42, SI-2000 Maribor, Slovenia; 5 Institute of Chemistry and Technology of Biobased Systems, Graz University of Technology, Stremayrgasse 9, AT-8010 Graz, Austria; karin.stanakleinschek@tugraz.at; 6 Laboratory for Characterisation and Processing of Polymers, Faculty of Mechanical Engineering, University of Maribor, Smetanova 17, SI-2000 Maribor, Slovenia

**Keywords:** 3D printing, wound dressings, alginate, carboxymethyl cellulose, polycaprolactone, polysaccharide-based scaffolds

## Abstract

Despite the extensive utilization of polysaccharide hydrogels in regenerative medicine, current fabrication methods fail to produce mechanically stable scaffolds using only hydrogels. The recently developed hybrid extrusion-based bioprinting process promises to resolve these current issues by facilitating the simultaneous printing of stiff thermoplastic polymers and softer hydrogels at different temperatures. Using layer-by-layer deposition, mechanically advantageous scaffolds can be produced by integrating the softer hydrogel matrix into a stiffer synthetic framework. This work demonstrates the fabrication of hybrid hydrogel-thermoplastic polymer scaffolds with tunable structural and chemical properties for applications in tissue engineering and regenerative medicine. Through an alternating deposition of polycaprolactone and alginate/carboxymethylcellulose gel strands, scaffolds with the desired architecture (e.g., filament thickness, pore size, macro-/microporosity), and rheological characteristics (e.g., swelling capacity, degradation rate, and wettability) were prepared. The hybrid fabrication approach allows the fine-tuning of wettability (approx. 50–75°), swelling (approx. 0–20× increased mass), degradability (approx. 2–30+ days), and mechanical strength (approx. 0.2–11 MPa) in the range between pure hydrogels and pure thermoplastic polymers, while providing a gradient of surface properties and good biocompatibility. The controlled degradability and permeability of the hydrogel component may also enable controlled drug delivery. Our work shows that the novel hybrid hydrogel-thermoplastic scaffolds with adjustable characteristics have immense potential for tissue engineering and can serve as templates for developing novel wound dressings.

## 1. Introduction

Dressings that accelerate wound healing should be biocompatible and antiseptic and they should retain moisture and enable oxygen and nutrient diffusion. They should also mimic the biochemical, mechanical, and topographical properties of the native extracellular matrix (ECM) [1], which should be optimized by adjusting polymer composition and concentration, as well as through the manufacturing procedure [2]. Furthermore, preventing transepidermal water loss during wound healing helps retain moisture in the wound and promotes the healing process during all stages [3]. A wound exposed to air dehydrates, which leads to scarring or the formation of a scab. Setting aesthetic issues aside, this presents problems because the formed mechanical barrier prevents epidermal cell migration and ultimately delays healing. Moreover, to facilitate the skin’s re-epithelialization, ensuring a moist environment is a prerequisite for successful wound management. In a moist wound, cells can move unimpeded through the thin layer of wound exudate, assisting in wound closure [4]. Nonetheless, it is important to stress that no single dressing is suitable for treating all wound types. For example, clean and granulated wounds require a moist environment, whereas in sloughy wounds, the main objective would be absorbing the exudate [3]. A wound dressing’s hydrophilic properties are related to its ability to absorb exudates, bacteria, and other impurities from the wound. Furthermore, concerning surface hydrophobicity, it is well known that this key parameter governs cell survival, responses, and communication [5]. Further, cell adhesion is unequivocally favored on more hydrophilic surfaces, compared with their more hydrophobic counterparts. On the other hand, hydrophobic surfaces prevent the binding of bacteria and might be a better option for drug delivery applications. All of these factors must be closely considered when designing wound dressing matrices [6].

Hydrogels are promising candidates for this purpose, as they (mostly) fulfill all the aforementioned requirements [7,8] by resembling native tissues in terms of composition and structure and in their ability to act as artificial ECM substitutes [9]. Their hydrophilic nature and soft tissue-like properties, combined with good nutrient/oxygen transportability, make them prime candidates for developing advanced wound dressings to treat large wounds, burns, and other skin lesions [10]. In particular, polysaccharide-based hydrogels are being popularized for cell-based applications because of their high biocompatibility, adjustable chemical and structural properties, low cost, and broad availability [11]. Alginate (ALG) and carboxymethyl cellulose (CMC) are among the most commonly employed polymers for the fabrication of wound dressings as they are non-toxic and allow good gelation control under mild conditions [12]. In soft tissue engineering, ALG is mainly utilized to improve mechanical stability and integrity of three-dimensional (3D) hydrogel constructs and to transmit mechanical signals to cells and developing tissues [13]. Mechanical properties of alginate hydrogels can be tailored through variations in composition sequence (i.e., mannuronic/guluronic acid (M/G) ratio), molecular weight (Mw), the total concentration of the polymer in the hydrogel, as well as by modifying the methods by which the hydrogel is post-processed (e.g., selection and concentration of the crosslinking agent, gelling time and temperature, as well as storage and incubation conditions of fabricated hydrogels) [14]. CMC is also an anionic water-soluble biopolymer derived from the chemical modification of cellulose. It demonstrates non-toxic and non-allergic properties and is widely used as a viscosity modifier or thickener or in wound dressings (e.g., Aquacel^®^) [15]. Like ALG, CMC contributes to cellular attachment and migration, and it might also promote wound healing [16].

By combining both polymers in different ratios, augmented characteristics can be obtained in one hybrid hydrogel formulation. The addition of ALG to CMC enhances the gelation mechanism, leading to marginally improved mechanical properties, which help to develop clinically relevant scaffolds [17]. The polymer mixture of ALG and CMC has been utilized in several published studies, as beads [18] or 3D bio-printed scaffolds [17] for controlled release and drug delivery. This polymer mixture seems favorable for 3D printing as it improves the solutions’ viscoelastic properties and gelation.

Despite the extensive utilization of polysaccharide hydrogels in tissue engineering and regenerative medicine, most fabrication methods have failed to produce stable 3D constructs and scaffolds using exclusively hydrogels [19], which are not suitable for maintaining the complex 3D architecture of the scaffold during or after the fabrication process [20]. Wound dressings made from natural polymers are therefore unable to maintain their long-term stability [21]. In contrast, thermoplastic polymers display favorable mechanical characteristics and maintain the required structural features. However, due to their poor solubility, they cannot provide the required highly hydrated environment for cells. Even more importantly, they often do not contain functional surface groups for promoting cell attachment. In order to improve biocompatibility, thermoplastic polymers require additional surface functionalization, whereas most hydrogels already contain required functional groups [22]. One of the most widely used thermoplastic polymers in regenerative medicine is polycaprolactone (PCL). PCL is a semi-crystalline biodegradable aliphatic polyester approved by the Food and Drug Administration (FDA) for biomedical applications [23]. Due to its biocompatibility and relatively high strength, it has found use in hard tissue engineering [24,25]. In addition, its great flexibility in synthesis and modification contributes to its popularity as one of the synthetic biomaterials for biomedical applications, including drug delivery and wound healing [26]. However, its slow biodegradability and lack of cell-binding sites and biomolecular signatures are important limitations to the range of potential applications [23]. Combining favorable attributes of thermoplastic polymers, such as PCL and hydrogels based on natural polymers, into hybrid scaffolds introduces novel applications and simultaneously increases the range of mechanical stability with bioactive properties [27]. For example, Kim and Kim demonstrated that hybrid PCL/alginate scaffolds with different alginate weight fractions exhibited better well-defined pore microstructures than pure ALG scaffolds. Hybrid scaffolds also showed significantly enhanced wetting behavior, water absorption, and increased biological activity than pure PCL scaffolds [28]. Moreover, some studies have shown that PCL fibers improve the mechanical stability of natural polymers (e.g., ALG, chitosan, gelatin) and facilitate cell attachment and proliferation [29].

Among many additive manufacturing techniques, extrusion-based 3D bioprinting is especially advantageous for fabricating tissue-regenerating scaffolds using a broad range of biocompatible materials [30]. This technology is particularly interesting for printing 3D tissue constructs that can be used for tissue regeneration purposes because it offers solid cell-laden freeform scaffolds with tailorable mechanical properties [31,32]. The main challenge in the development of these techniques is maintaining the overall mechanical integrity and shape fidelity of the constructs during printing or cell culture conditions [33]. The possibility of a so-called “hybrid” approach of bioprinting that combines stiff thermoplastic polymers and softer hydrogels promises to resolve the abovementioned issues [27,34].

We hypothesized that by using a hybrid 3D extrusion bioprinting process, we could fabricate scaffolds with tunable mechanical and physicochemical properties in terms of porosity, stiffness, swelling, and degradation. Such scaffolds would be suitable for advanced tissue engineering applications and the design of novel wound dressings.

## 2. Materials and Methods

### 2.1. Materials

The sodium salt of alginic acid (ALG, Mw: 80 kDa, CAS num.: 9005-38-3), carboxymethyl cellulose (CMC, in the form of sodium salt, Mw: 700 kDa, degree of substitution of 0.9, CAS num.: 9004-32-4), polycaprolactone (PCL, Mw: 45 kDa), and other chemicals (CaCl_2_) for scaffold production were all purchased from Sigma-Aldrich, Germany. All materials and chemicals were used as purchased without any further modification. For the preparation of all solutions and experiments, ultra-pure water (18.2 MΩ·cm at 25 °C) was used, prepared using an ELGA Purelab water purification system (Veolia Water Technologies, High Wycombe, UK).

### 2.2. Preparation of 3D Printed Scaffolds

To be suitable for further processing and analysis, the deposited material had to hold its own weight and allow the preparation of structures with 10 or more layers that retain their shape (height, filament thickness, and pore size) throughout the fabrication process. The basic ‘ink’ formulation to fulfill the mentioned requirements was a hydrogel of 5 wt.% ALG, 5 wt.% CMC, further stabilized by adding 3 g of 5 wt.% CaCl_2_ solution to 100 g of gel, which could then be extruded directly at ambient conditions (AC ink). As an alternative ‘ink’ (P ink), pure PCL was used, which was melted at 80 °C, followed by fused deposition 3D printing at the mentioned temperature. PCL-based scaffolds solidified again at room temperature, retaining their printer form without further post-processing.

Four types of scaffolds were prepared for further testing and analysis: AC scaffolds, AC scaffolds with additional crosslinking (ACC—additional scaffold soaking in 5 wt.% CaCl_2_ for 15 min at room temperature), P scaffolds (printed using pure PCL), and ACCP (hybrid) scaffolds. ACCP scaffolds were produced with the simultaneous use of two extruders by depositing alternating layers of ACC (using one extruder) and PCL (using the second extruder). The finished scaffolds were cross-linked with 5 wt.% CaCl_2_. Structure design and slicing were performed using Autodesk Fusion 360 (Autodesk Inc., Mill Valley, CA, USA) and Slic3r v1.2.9 software, with a rectilinear infill pattern. Cuboid and cylindrical scaffolds were prepared, along with additional geometries to comply with tensile testing (prepared according to ISO 527-2:2012 type 1BB). All scaffolds were prepared using the BioScaffolder 3.1 (GeSiM, Germany) bioprinter and conical, blunt-end extrusion nozzles (Nordson EFD, Westlake, OH, USA) with an inner diameter of 0.25 mm. All manufactured scaffolds retained their structural stability and preserved shape fidelity after deposition of 50 subsequent layers, as well as prolonged periods in cell culture conditions (soaked in Advanced Dulbecco’s Modified Eagle’s Medium (ADMEM) at 37 °C and 5 wt.% CO_2_).

### 2.3. Scaffold Characterization Methods

#### 2.3.1. Morphology Assessment of the 3D Printed Scaffolds Using Optical Microscopy

To optimize the scaffold composition and the 3D printing procedure, as well as to morphologically assess the scaffolds, all intermediate printed scaffolds were observed under a fluorescence microscope (EVOS, FL Cell Imaging System, Thermo Fisher Scientific Inc., Waltham, MA, USA) using magnifications of 2×, 4×, and 10× in bright field (BF) mode, respectively. To evaluate the printed filament thickness and the scaffold macropores’ sizes, freely available NIH image analysis software ImageJ 1.52 g (NIH, Bethesda, MD, USA) [35] was used. The result is reported as the average measurement from at least thirty measurements with the standard error for each measurement.

#### 2.3.2. Atomic Force Microscopy

The topographical and surface roughness analysis of all optimized formulations was performed with atomic force microscopy (AFM) in tapping mode (Keysight 7500 AFM multimode scanning probe microscope, Keysight Technologies, Wokingham, UK). Samples for AFM measurements were prepared by pressing the formulations between two silicon wafers, which were then removed, and the samples were appropriately post-processed. The images were scanned using silicon cantilevers (ATEC-NC-20, Nanosensors, Neuchatel, Switzerland) with a resonance frequency of 210–490 kHz and a force constant of 12–110 Nm^−1^. All measurements were performed at room temperature. For all samples, images of 10 × 10, 5 × 5, and 1 × 1 μm^2^ were recorded with a resolution of 1024 × 1024 pixels [36]. Pico Image Basic 7.2 software (Keysight Technologies, Wokingham, UK) was used to process all images and to calculate the root mean square height of the surface (S_q_) and arithmetical mean height of the surface (S_a_) according to ISO 25178.

#### 2.3.3. Scanning Electron Microscopy

The surface morphology and porosity of all final 3D printed scaffolds were analyzed by scanning electron microscopy (SEM) [37]. Before imaging, all freshly 3D printed scaffolds were frozen at −80 °C, lyophilized, and pressed on a double-sided adhesive carbon tape (SPI Supplies, West Chester, PA, USA). For obtaining cross-sectional images, lyophilized scaffolds were cut longitudinally using a scalpel. Thereby we were able to assess the inside porosity of each sample. Micrographs were taken using a field emission scanning electron microscope (FE-SEM, Supra 35 VP, Carl Zeiss, Oberkochen, Germany) operated at a low accelerating voltage (1 keV) and room temperature. Images were acquired at a working distance of 4.5–5.5 mm at magnifications 2500 and 10,000, respectively.

### 2.4. Applicative Properties of 3D Printed Scaffolds

#### 2.4.1. Wettability

The prepared 3D printing inks’ wettability was determined using an OCA15+ goniometer system (Dataphysics, Filderstadt, Germany) with the sessile drop method [36]. Flat samples for wettability measurements were prepared by pressing the formulations between two silicon wafers, which were removed after the formulation solidified. To create flat hydrogel/PCL surfaces (mimicking the hydrogel/PCL interface), we poured the molten thermoplastic polymer into the hydrogel and pressed the formulation between silicon wafers until it solidified. Static contact angle (SCA) measurement was carried out using ultra-pure water at ambient temperature. All measurements were carried out on at least three independent sample surfaces with a drop volume of 2 µL. Each SCA value was the average of at least six drops of liquid per sample surface.

#### 2.4.2. In Vitro Swelling Test

The swelling kinetics of the scaffolds was investigated by the gravimetric method [38]. Firstly, all freshly printed scaffolds were dried for 3 days at room temperature and then weighed (initial weight, W_0_). The dry scaffolds were immersed in phosphate-buffered saline (PBS) (pH 7.4) at 37 °C. At predetermined time intervals, the sample was removed from the PBS, wiped dry with filter paper to remove excess liquid from the surface, and weighed. The swelling ratio at time t was calculated using Equation (1):

Swelling ratio (%) = (W_t_−W_0_)/W_0_ × 100%, (1)

where W_0_ and W_t_ are the weights of the initial dry scaffolds and swollen scaffolds at fixed time intervals t, respectively. All measurements were executed in triplicate, and the final result is reported and plotted as the average with the corresponding standard error.

#### 2.4.3. In Vitro Degradation Test

Before the start of the in vitro degradation test, according to [39], freshly prepared cube-shaped scaffolds (1 cm^3^) were dried for 3 days at room temperature and then weighed (W_0_). Subsequently, dry scaffolds were placed in plastic flasks with 10 mL PBS (pH 7.4) at 37 °C and, at predetermined time intervals, the specimens were removed from PBS, washed with deionized water three times, and dried again. Separate samples were prepared for measurements at each degradation time point, so that sample handling was minimized. The weight remaining of the scaffolds was calculated according to Equation (2):

Weight remaining (%) = W_t_/W_0_ × 100%, (2)

where W_0_ is the initial weight of the dry scaffold and W_t_ is the weight of the scaffold at fixed time intervals. Again, all experimental data were obtained from triplicate samples, and the result is reported and plotted as the average with the standard error.

#### 2.4.4. Mechanical Properties—Compression and Tensile Test

The 3D printed scaffolds’ mechanical properties were defined using quasi-static uniaxial compression and tensile tests, using a Tinius Olsen H10KT compression/tension testing machine (Tinius Olsen TMC, Horsham, PA, USA) [40]. Test specimens of the scaffolds were 3D printed as described in Section 2.2 on the day of testing to preserve the quality of the materials’ mechanical behavior. The specimen size and shape were determined based on ISO 527-2:2012 type 1BB for the tension test and as cylinders (diameter: 30 mm, height: 3 mm) for the compression test because cylinder shapes are more commonly used for plastic testing. The size and shape of the used standard allowed for the feasible manufacturability of the specimens. Test measurements were performed for each material to define the magnitude of the force and resulting deformation. Based on the preliminary results, the optimal settings for actual measurements were determined. We used 50 N, 1000 N, and 10,000 N load cells for optimal resolution in terms of the resulting force. For the actual measurements, three specimens were tested and checked for repeatability.

#### 2.4.5. Viability of Skin Cells

All cell tests were conducted using human skin-derived cells; namely, commercially available skin fibroblasts (ATCC CCL-110, Detroit 551, LGC Standards, Teddington, UK) and an aneuploidy immortalized keratinocyte cell line from adult human skin (HaCaT). The latter were kindly provided by Prof. Dr. Elsa Fabbretti (Centre for Biomedical Sciences and Engineering, University of Nova Gorica, Slovenia). The influence of different 3D printed scaffolds on cell viability was evaluated via the reduction reaction of the tetrazolium salt MTT (3(4,5 dimethylthiazolyl-2)-2,5-diphenyltetrazolium bromide), purchased from Sigma Aldrich, Germany. This is a widely accepted and reliable method to examine cell viability through cellular metabolic activity [41]. The MTT assay measures cellular metabolic activity as an indicator of cell viability, proliferation, and cytotoxicity. Viable cells contain NAD(P)H-dependent oxidoreductase enzymes that reduce MTT to formazan, resulting in a colored solution after its dissolution that can be measured and quantified. Thus, the greater the number of viable, metabolically active cells, the darker the solution; conversely, cell viability is reduced when metabolic processes lead to apoptosis or necrosis, as indicated by the lighter color of the solution [42]. In our case, the MTT cell viability assay was performed according to Mosmann [43]. The sample extraction was carried out according to ISO-10993-5 and ISO 10993-12 regulations (10993-5 AAI. Biological evaluation of medical devices–Part 5: Tests for in vitro cytotoxicity. 2009.10993-12 I. Biological evaluation of medical devices–Part 12: Sample preparation and reference materials. 2007). Briefly, freshly 3D printed cylinder-shaped scaffolds (diameter 10 mm; height 5 mm) were sterilized under ultraviolet (UV) light for 30 min and soaked into 3 mL of Advanced Dulbecco’s Modified Eagle’s Medium (ADMEM; Thermo Fisher, Waltham, MA, USA) supplemented with 5 *v*/*v*% Foetal Bovine Serum (FBS; Thermo Fisher Scientific Inc., Waltham, MA, USA), and incubated for 24 h at 37 °C in an atmosphere containing 5 wt.% CO_2_. The HaCaT and skin fibroblast cells (10,000 cells/well) were seeded into a 96-well microtiter plate with a final volume of 100 μL of ADMEM medium supplemented with 5 *v*/*v*% FBS. All cells were exposed to the same volume (100 μL) of the as-prepared respective sample solutions, and their dilutions of 1:2, 1:4, 1:8, and 1:16, followed by incubation for 24 h at 37 °C in an atmosphere containing 5 wt.% CO_2_. All experiments were performed in four parallels. Spectrophotometrical detection of the color change was measured using the Varioskan multiplate reader (Thermo Fisher Scientific Inc., Waltham, MA, USA) [44]. Final results are shown as average values with corresponding standard deviations.

### 2.5. Statistical Analysis

All numerical values are reported as mean ± standard deviation (SD). The Shapiro–Wilk test confirmed the normal distribution of the experimental data. Levene’s test was used to assess the equality of variances. As all data sets were well-modeled by a normal distribution and homoscedastic, one-way analysis of variance (ANOVA), followed by the Bonferroni post-hoc test, was carried out accordingly. Obtained *p*-values < 0.05 were considered statistically significant. Statistical analysis was performed using SPPS Statistics 27 (IBM Corp. Armonk, NY, USA).

## 3. Results and Discussion

Reproducible 3D printing of biocompatible and structurally stable hydrogels still represents a challenge [20]. Improving hydrogels’ biocompatibility and functionality is usually accomplished by surface modification of the material. This is often performed to maintain or even improve its properties regarding stability, degradation resistance, hydrophobicity, or other features based on specific applications [6]. Herein, we hypothesized that an alternative approach using an optimized hydrogel formulation as the basis, integrated with a synthetic PCL-based ink, could enable the 3D printing of scaffolds with tunable mechanical and physicochemical properties (e.g., degradation, swelling, topography). As thoroughly explored and discussed in the upcoming sections, the physicochemical (e.g., swelling, degradation) and biological features of the fabricated scaffolds are mainly governed by material characteristics (the scaffold’s building blocks) and the 3D construct’s design [45].

### 3.1. Formulation Preparation and 3D Printing of Hybrid Scaffolds

Reproducible 3D printing of scaffolds requires homogeneous coextrusion of both the hydrogel and the synthetic component, without crossover or retraction movements, which could interrupt the flow. The macropore size (distance between filaments) was precisely modeled in the g-code by considering the extruded filament’s outer diameter and the distance between the gridlines. Both the scaffold computer-aided design (CAD) model and the set macropore size (0.7 × 0.7 mm) were identical across all scaffolds (except for mechanical tests, which required specific specimens), regardless of the composition. Because of the extensive utilization of polysaccharide polymers as the main components in various formulations for wound treatment, the ALG and CMC polymer mixture was chosen as the hydrogel base material. Comparative studies showed that CMC possesses the ability to absorb harmful bacteria and, additionally, ALG likewise exhibits antimicrobial activity, although to a lesser extent than CMC [46]. Generally, mixing ALG with CMC increases the solution’s viscosity, which aids the gel’s fast formation during fabrication [47]. Fast gelation is one of the pivotal requirements for an extrusion-based printing system. On account of the carboxyl (-COOH) groups present in ALG and CMC, a solution of 5 wt.% CaCl_2_ was used as a chemical crosslinking agent. Its addition further improved the formulation’s viscoelastic properties. The printing of this formulation yielded the AC scaffolds. Other divalent cations (Mg^2+^, Sr^2+^, Br^2+^) or solutions containing calcium (CaSO_4_, CaCO_3_) can be used for ionic crosslinking. However, CaCl_2_ yields the fastest gelation rates and displays the highest crosslinking efficiency [48]. Aside from forming ionic bonds with two functional groups simultaneously and forming connections between polymer chains, resulting in increased rigidity of the hydrogel construct, the addition of Ca^2+^ might be useful in all stages of the wound healing process. When released into the wound, Ca^2+^ ions may improve some cellular aspects of wound healing (e.g., increased fibroblast proliferation, activation of human macrophages) and aid the clotting mechanism during the first stage of the wound healing process [3]. Due to a lower crosslinking level (since CaCl_2_ was only added into the formulation and was not used for post-processing), the AC scaffolds failed to maintain their structural integrity in the cell culture medium at 37 °C. In comparison, the completely crosslinked ACC scaffolds (i.e., these scaffolds were post-processed with CaCl_2_) proved stable throughout the incubation cycle (72 h), as did the P and ACCP scaffolds.

**Figure 1:**
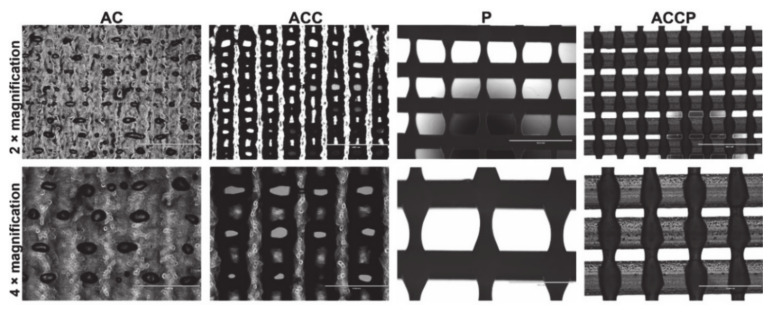
Macropores of 3D printed scaffolds visualized under 2× and 4× magnification.

Geometrical characterization was performed by means of visual assessment of the fabricated structures’ geometry using an optical microscope (EVOS, FL Cell Imaging System, Thermo Fisher Scientific Inc., Waltham, MA, USA). The printed layers of all scaffolds (AC, ACC, P, and ACCP) were evaluated regarding macropore size (pore length, width, and area) and filament thickness, and the construct’s overall macroporosity was calculated. The obtained images of the printed scaffolds’ meshes are presented in Figure 1. Corresponding results of the measurements are shown in Table 1.

A pore is defined as a void space within a scaffold. In contrast, porosity, a morphological property independent of the material, can be considered a collection of pores and represents the percentage of empty space within a solid. The pore size and degree of porosity are also crucial in facilitating cell migration, proliferation, and vascularization. Macropores (pore size > 50 µm) facilitate nutrient distribution, enhanced oxygen diffusion, and improved catabolite removal [49]. Due to cell size, migration requirements, and transport, pore sizes of approximately 100 μm are considered to be the minimum requirement. However, due to enhanced tissue (skin) formation and the formation of capillaries, pore sizes above 150 μm are generally recommended [50]. Materials with lower porosity suppress cell proliferation and force cell aggregation. On the contrary, highly porous matrices are beneficial in wound dressings for several reasons, including promoting moisture retention, preventing infection, and facilitating the transport of nutrients and oxygen [21]. High porosity also contributes to cellular infiltration and tissue ingrowth. However, increasing macroporosity reduces the mechanical stability of the material. A balance in pore size and porosity must be set, depending on the new tissue formation rate and the scaffold material’s degradation rate.

Since the geometrical parameters were constant across all scaffolds in this work, the influence of different base formulations and the significance of crosslinking on the meshes of the resulting printed scaffolds was undoubtedly evident. By comparing the obtained images (Figure 1) and measured parameters (Table 1), a variable filament thickness, macropore size, and shape can be observed for all scaffolds. The differences in measured parameters can be ascribed (among other factors) to the differences in viscosity of the base polymers and, in the case of AC and ACC scaffolds, to a different post-processing regime [51]. Due to its relatively high melting point and rapid cooling upon deposition, the PCL (P) scaffold manufacturing allowed the highest degree of filament and pore uniformity. The P samples’ filaments were the thinnest (closest to the inner nozzle diameter), and consequently their macropores were the largest. In turn, P scaffolds also possessed the highest macroporosity (56.78%), which is well suited for the fabrication of advanced wound dressings.

On the contrary, the hydrogel formulation’s low viscosity limits the fabrication of stable constructs, as is evident from the poorly defined filaments and pores of the AC scaffolds. Expectedly, both AC and ACC scaffolds displayed a significant increase in filament thickness, a decrease in macropore size, and a more significant variation in pore size and shape compared to P scaffolds. Among all results, the AC scaffolds displayed the thickest filaments, smallest macropores, and consequently the lowest macroporosity (1.53%). In comparison to AC scaffolds, their ionically crosslinked counterparts displayed a significant decrease in filament thickness and an increase in pore size. Furthermore, this also resulted in a nearly 10-fold increase in macroporosity (9.73%) and a more uniform pore size and shape. We showed that the inclusion of this simple post-processing step significantly improves the hydrogel scaffold’s mechanical characteristics and preserves its 3D-printed architecture.

Considering the “softer” nature of hydrogels and the synthetic PCL’s favorable mechanical attributes, intermediate morphological features were expected when combining all the polymers into one scaffold. The hybrid ACCP lattice indeed exhibited intermediate macropore size and filament thickness. It is important to note that the pore length and width of hybrid scaffolds differed significantly, resulting in a seemingly more variable filament thickness. This is a consequence of significantly thicker hydrogel filaments (in agreement with AC and ACC scaffolds) and substantially narrower PCL filaments (similar to P scaffolds) being combined in one construct. Despite the low viscosity of the used AC hydrogel, the alternating AC-P lattice structure remained stable. More importantly, the hydrogel filaments did not fuse and no macropore closing was observed, arguably due to the mechanical support provided by the PCL filaments. By serving as a framework, PCL helps retain the scaffold’s printed geometry and results in the higher macroporosity of the hybrid construct (15.72%) compared to the hydrogel scaffolds. ACCP scaffolds also enable ionic crosslinking of the hydrogel constituent through post-processing, which has no noticeable effects on the synthetic component. Modulating the ratio of the ACCP hybrid scaffolds’ components presents the opportunity to tailor the geometrical properties of scaffolds, which influence their mechanical behavior. This is examined below. The highly microporous hybrid 3D construct is more beneficial for supporting the healing process through all stages than the “pure” hydrogel structures [52].

To maintain the scaffolds’ long-term structural integrity, some crucial parameters of the hybrid biofabrication process have been established. When dispensing the thermoplastic PCL and a hydrogel mixture (e.g., ALG and CMC), all materials’ rheological properties should be considered. According to the literature [48], the filament width, fabrication time, and printing resolution are influenced by the extruded material’s flow rate. The latter is directly proportional to the extrusion pressure and inversely proportional to the viscosity of the material. For well-controlled deposition of materials with varying viscosities, precise adjustment of the extrusion pressure is required over a range of pressures. However, as a function of temperature, material viscosity can also be adjusted, although with limitations when using bioinks. In the fabrication of ACCP scaffolds in this work, PCL temperature was adjusted to 80 °C to match the AC hydrogel’s viscosity. This reduced the required extrusion forces, ensured the precise flow of both components, and facilitated the high-resolution fabrication of hybrid scaffolds. During the fabrication process, higher PCL extrusion temperatures did not present additional issues for dispensing hydrogel layers. The thinness of the deposited PCL strands enabled the rapid cooling of filaments, which mechanically supported the deposition of hydrogel strands. By optimizing the base solution’s viscosity and regulating the extrusion pressure and temperature, retraction speeds, and the distance between the substrate and the nozzle, it was possible to 3D print reproducible hybrid 3D constructs with controlled geometry and fully interconnected pores.

### 3.2. Scaffold Surface Properties and Morphology

The material’s surface characteristics (e.g., roughness) and morphology are important factors affecting cell adhesion. Through the physicochemical interaction between the cells and the substrate, cytoskeletal re-arrangement in cells is prompted, influencing migration, proliferation, and differentiation [53]. However, it is important to note that the cells’ response to surface properties differs depending on cell type [54].

**Figure 2:**
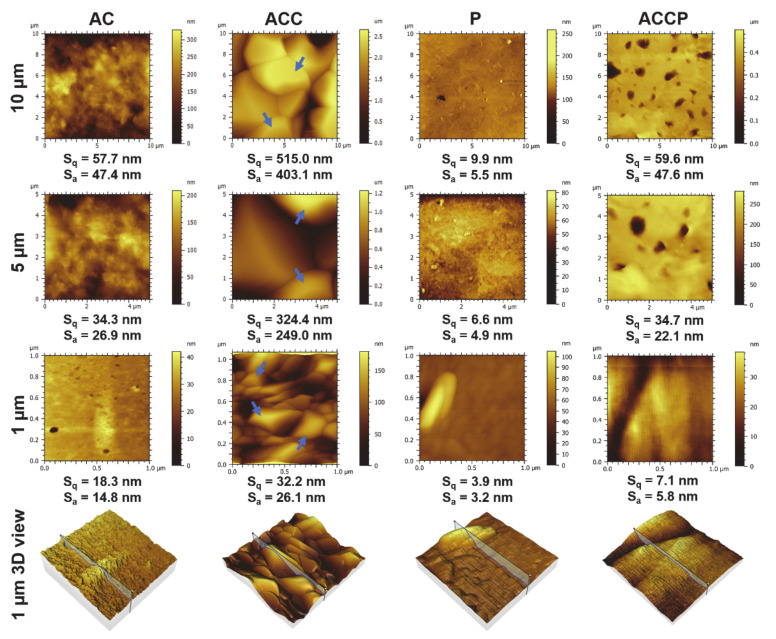
Scaffolds’ surface morphology and roughness parameters as measured by atomic force microscopy. The arrows indicate CaCl_2_ crystals on the surface of crosslinked alginate and carboxymethyl cellulose (ACC) scaffolds. P, pure polycaprolactone scaffold; ACCP, scaffold with alternating layers of ACC and PCL.

The surface characteristics, morphology, and roughness of all formulations and the influence of ionic crosslinking on hydrogels and different material combinations (hydrogel + PCL) on the mentioned features were evaluated by performing AFM measurements directly on the formulations. The surface morphology and corresponding surface roughness parameters S_q_ and S_a_ for respective scaffolds at 10 μm, 5 μm, and 1 μm scales are presented in Figure 2.

For AC, P, and ACCP samples, easily observable surface features were visible regardless of the image size. Based on the AFM micrographs and the calculated roughness parameters, it was evident (and expected) that the PCL-only scaffolds (P) exhibited the overall flattest and smoothest surface morphology. On the other hand, the AC formulation building blocks (ALG/CMC) were larger. Thus, it was anticipated that hydrogel scaffolds would display significantly rougher topography than P scaffolds [55]. This can be attributed to differences in size, orientation, and organization between ALG/CMC and PCL polymer chains present on the formulations’ surface. The AC scaffold’s calculated roughness parameters supported this statement as they were approximately 5× higher on all size scales compared to their synthetic (PCL) counterparts. No larger polymer aggregates were observed for both P and AC scaffolds regardless of the size scale of the measurement. Interestingly, based on the images, it was obvious that the post-processing step significantly affected the ACC hydrogel scaffolds’ surface morphology. By ionically crosslinking scaffolds after 3D printing, a nearly 10-fold increase in ACC scaffolds’ surface roughness parameters was observed, compared to AC. This increase in surface roughness can be attributed to two key factors. Analyzing only the AFM scans of ACC scaffolds and comparing them to the AC ones, larger features were clearly visible at all size scales. This can most likely be ascribed to excess CaCl_2_ deposits on the scaffolds’ surface. During the drying procedure, CaCl_2_ left over on the surface forms crystals, which can be clearly observed on the AFM images. The second factor, contributing to an overall increase in scaffold roughness, is the effect of crosslinking on the hydrogel formulation’s molecular nature. As shown in the results of optical (bright-field and fluorescence) microscopy, scaffold filaments of the post-processed ACC scaffolds were significantly finer than the untreated AC filaments, suggesting a denser polymer network in ACC scaffolds, which was also confirmed by SEM (see below). Apart from a rougher topography, the AFM images of ACCP scaffolds also show a less homogenous surface compared to the P scaffolds. Especially at the smallest size scale (1 μm), ACCP scaffolds exhibited surface characteristics that could be best characterized as an intermediate between the apparent homogeneous surface of P scaffolds and the particle-like attributes of the AC scaffold surface. This suggests that we can tailor the scaffolds’ roughness by adjusting the hydrogel composition and through post-processing within a hybrid scaffold.

**Figure 3:**
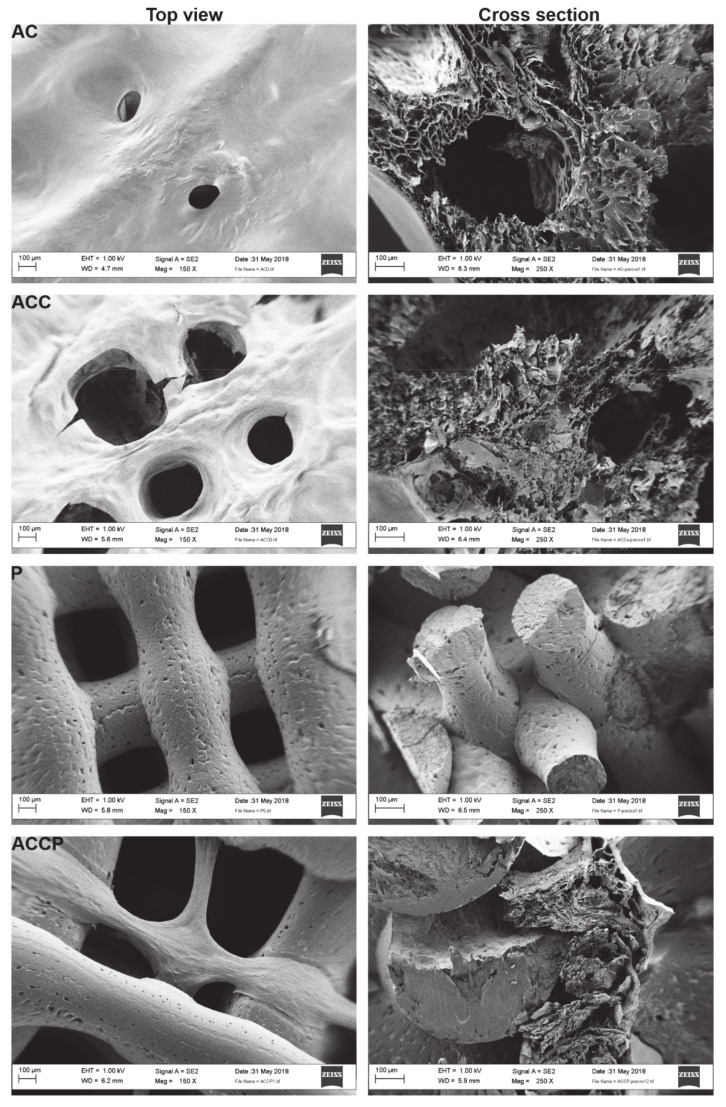
SEM images of the 3D printed scaffolds.

AFM’s main limitation is that it only provides topological information on a relatively small part of the scaffold’s surface (even if the test is repeated at multiple discrete locations on the sample). Therefore, SEM was used as a complementary technique to analyze the scaffold’s surface morphology and observe the influence of different material combinations and ionic crosslinking on scaffolds’ micro-geometry. By cutting the lyophilized scaffolds longitudinally, the scaffolds’ internal microporosity was also assessed. Figure 3 shows the results of SEM analysis. Scaffolds’ surface micrographs (top view) were taken at 150× magnification and cross-sectional micrographs were taken at 250× magnification.

The results obtained from SEM imaging were consistent with observations made using optical microscopy in terms of pore size and filament thickness. As the printing parameters (except for the printing temperature of PCL) and pore size were set as a constant for the fabrication of all scaffolds, it was evident that the scaffolds’ micro-geometrical characteristics and surface morphologies primarily depended on the material selection. For AC and ACC scaffolds, which differed only in the post-processing regime, significant size differences in pores and filaments were noticeable. Soon after 3D printing, AC scaffolds lose their 3D printed architecture, do not retain shape fidelity, and the scaffold’s filaments are prone to fusing, causing macropores to lose their set size and shape. On the other hand, crosslinked AC (ACC) scaffolds are superior in retaining their 3D structure and predefined macropore size and geometry and display significantly thinner filaments that do not fuse. In the case of ACC scaffolds, it is also important to point out that no larger CaCl_2_ crystals are visible on the scaffolds’ surface. Combined with AFM results, this suggests that only a thin layer of CaCl_2_ is uniformly distributed on the scaffolds’ surface. By examining the cross-sectional images, the scaffolds’ interior microporosity was assessed. Sufficient scaffold microporosity is considered beneficial. However, high microporosity can negatively influence the scaffold’s structural and degradation properties [56]. The SEM images confirmed that AC scaffolds exhibited a more microporous inner structure overall, in comparison to ACC scaffolds. The latter has a more homogenous and densely compacted inner framework, which is, as previously alluded to, a consequence of the additional ionic crosslinking of ALG/CMC. The tighter AC hydrogel inner architecture also implies a more rigid molecular structure, ultimately leading to a more mechanically favorable scaffold. As discussed below in Section 3.3.2 (In Vitro Swelling and Degradation Tests, a tighter crosslinked network and lower microporosity greatly improve the ACC scaffold’s swelling and degradation properties.

In contrast, P scaffolds display virtually no inner microporosity. Smaller indentations on the PCL scaffold’s surface and in the filament’s core (which are also evident in AFM images of P and ACCP scaffolds) are probably a consequence of trapped air bubbles, which tend to form during 3D printing of PCL at higher temperatures. P scaffolds display a more uniform filament thickness, pore size, and pore geometry than hydrogel scaffolds. Even in the cross-sectional micrographs, the filaments’ shape and inner structure are uniform. The main advantage of P scaffolds is that they maintain their shape fidelity and 3D printed geometry after the fabrication procedure. This makes PCL a great framework for supporting a softer “ECM-mimicking” hydrogel component. Indeed, that is what we observed in the SEM images of hybrid ACCP scaffolds. Mechanically advantageous PCL supports hydrogel filaments and prevents filament collapse and fusion. Therefore, hybrid scaffolds can retain their 3D printed architecture (e.g., pore size and shape, filament thickness). The construct’s overall microporosity can be improved by introducing a microporous hydrogel component into the impermeable PCL.

### 3.3. Determination of General Wound Dressing Performance-Related Properties of 3D Printed Scaffolds

#### 3.3.1. Hydrophilicity

Wound dressings are generally evaluated in terms of their water uptake capacity, whereas the sample’s hydrophilicity presents the main measured characteristic. A common method for assessing the latter is measuring the water contact angle CA(H_2_O), which is correlated with the material’s hydrophilicity. Generally, CA(H_2_O) values are inversely proportional to water retention. However, it is important to note that the results obtained on a two-dimensional (2D) surface cannot be fully extrapolated to a 3D construct straightforwardly. The scaffold’s 3D structure on all size scales is also a key parameter in regulating its overall hydrophilicity, especially in synthetic polymers and fibrous materials [57]. The CA(H_2_O) values for all our samples are shown in Table 2. The results are presented as averages of at least six measurements per surface, with corresponding standard deviations (as described in Section 2).

As polysaccharide-based hydrogels generally show increased hydrophilicity compared to synthetic materials (e.g., PCL), it was expected that both hydrogel-only formulations (AC and ACC) would exhibit the most hydrophilic properties among all formulations. Indeed, the values obtained for these two formulations were CA(H_2_O) = 13.7 ± 1.6° for ACC and CA(H_2_O) = 50.0 ± 3.8° for AC. The obtained values can most likely be ascribed to differences in surface morphology and increased surface roughness compared to synthetic polymers, and the high content of –OH and –COOH groups present in both polymers (ALG and CMC). These increase AC and ACC scaffolds’ surface affinity to water and contribute to hydrogel’s hydrophilic properties. According to the literature, pure ALG’s hydrophilicity depends on the polymer form and can vary from 54.0° to 90.0° [3]. The water contact angle for pure CMC is significantly lower and was measured to be between 20.0° and 30.0° [58]. Therefore, our obtained CA(H_2_O) = 50.0 ± 3.8° for the AC scaffold is expected and consistent with the literature.

The results show that the post-processing regime and the choice of crosslinking agent affect the hydrogel formulation’s hydrophilic nature. By additionally crosslinking the AC scaffolds with Ca^2+^ ions, we were able to significantly increase the surface hydrophilicity. Altered surface characteristics can explain this phenomenon (e.g., surface roughness and homogeneity) observed in ACC scaffolds, as well as the presence of CaCl_2_ on their surface. If we take the AFM surface roughness parameters into account, the most likely explanation for this increased hydrophilicity is an overall increase in surface roughness. A nearly 10-fold increase in surface roughness parameters was observed by crosslinking the scaffolds, which indicates a rougher surface compared to AC scaffolds. It is well established that specific surface morphology (e.g., high micro/nano roughness) significantly impacts a surface’s wettability [59]. Therefore, we can assume that the reduction of CA(H_2_O) is likely to reflect the rougher and less homogenous surface of ACC scaffolds. This result is of great importance because it shows that we were able to easily modulate the hydrogel’s hydrophobic/hydrophilic properties by changing the post-processing regime or the crosslinking agent.

For pure PCL (P scaffolds’ CA(H_2_O) = 75.9 ± 0.9°), which is known for its hydrophobic nature, the highest contact angle among all tested materials was also anticipated. The ALG/CMC hydrogel addition into the PCL matrix reduced the CA(H2O), pointing to an incremental increase in the hybrid formulation’s hydrophilicity. The mixture of all three base materials (ALG/CMC/PCL) displays an intermediate CA(H_2_O) = 66.9 ± 2.2°, which lies between the AC and P scaffolds. This value drop demonstrates that incorporating polymers with a hydrophilic character (e.g., ALG/CMC) into the synthetic PCL framework presents a practical strategy for tailoring hydrophilic/hydrophobic properties of 3D printed constructs. Most of them, especially modern wound dressings, are hydrophobic, which, coupled with their exudate absorption, is of great importance in the elimination of passive bacteria [3]. We showed that by additionally crosslinking the hydrogel and changing the hydrogel component’s overall mass ratio in the PCL framework, we could, according to specific needs, easily tailor the hydrophilic properties of scaffolds. Owing to PCL’s hydrophobic character and the ALG/CMC component’s highly absorptive ability, the hybrid ACCP formulation presents itself as the most promising material for the further development of advanced wound healing matrices.

#### 3.3.2. In Vitro Swelling and Degradation Tests

When designing scaffolds for wound dressings, one of the crucial parameters to consider is their water absorption ability. A hydrogel’s swelling ratio may be associated with increased diffusion of signaling molecules and nutrients into the material. Hence, scaffolds with higher water absorption capacity might facilitate the transportation of nutrients and metabolites into a wound. Many factors, such as the material’s physicochemical properties, the types of crosslinking agents used, the overall crosslinking density, and the presence of hydrophilic groups, influence the swelling rate [60].

**Figure 4:**
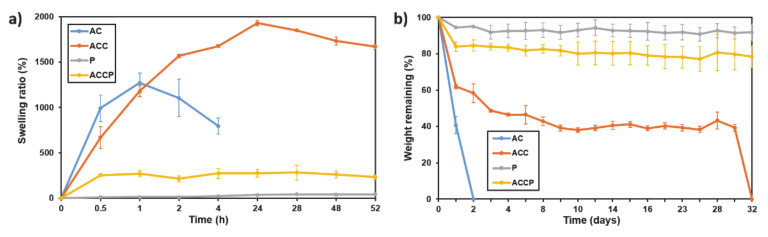
(**a**) Scaffold swelling test. (**b**) Scaffold degradation test.

Another fundamental property that needs to be considered when designing scaffolds for wound dressings is their degradation rate. The scaffold should simultaneously be stable enough to allow native cells to populate its surface/bulk and degrade at a suitable speed for the controlled release of bioactive molecules or allow for the subsequent native tissue regeneration. In general, hydrogel scaffolds’ mechanical stiffness is proportional to their degradation rate, as both properties show a linear correlation with overall crosslinking density [61]. All the tested scaffolds’ swelling and degradation rates were monitored by measuring changes in their weight over time when incubated in PBS at 37 °C. Significant differences in swelling and degradation rates among various formulations were observed, as illustrated in Figure 4.

Figure 4a shows that both hydrogel-only (AC and ACC) scaffolds display an upward trend in swelling rate over time. However, the AC scaffold reached its apparent maximum swelling capacity within 1 h, followed by a rather quick scaffold degradation. This effect is probably partially the consequence of handling the fragile samples to weigh them at defined time intervals. The AC scaffolds reached a maximum swelling rate of 1270% of their initial weight, which is consistent with previous studies [62]. However, it is obvious from Figure 4 that the scaffolds’ degradation speeds quickly overtook their ability to absorb water further. On the other hand, the post-processed ACC scaffolds needed approximately 24 h to reach their swelling equilibrium and only started to degrade slowly after 1 day of incubation in PBS. The swelling kinetics of the AC and the ACC scaffolds appeared similar for the first 1 h of the test. However, the ACC constructs continued to absorb water and swelled to nearly 2000% of their initial weight. Despite the apparently similar initial swelling kinetics, the AC scaffolds seemed to swell faster. As depicted in Figure 4b, the weight of hydrogel-only scaffolds (AC and ACC) significantly decreased over prolonged incubation periods. For the AC construct, the fastest degradation rate was observed. After 24 h the AC scaffolds lost more than 60% of their initial weight, followed by their total degradation after 48 h. On the contrary, the ACC scaffolds’ weight quickly dropped in the first 48 h to 60% of their initial dry weight and then slowly declined over 30 days. These results imply that the differences in swelling and degradation speed can be attributed to the scaffolds’ changed molecular nature due to post-processing with CaCl_2_. Both AC and ACC were crosslinked by applying divalent cations (Ca^2+^); however, they differed in the post-processing step, in which the ACC scaffolds were also fully crosslinked. Additional application of Ca^2+^ ions forms extra ionic bonds with two carboxyl groups simultaneously, forming connections between polymer chains. This increases the polymer network’s rigidity and density and decreases the hydrogel’s microporosity and consequently hinders water diffusion into the construct [63]. The overall increased crosslinking density resulted in a structurally more stable ACC scaffold (less microporous), leading to slower water absorption. In contrast, the overall lower crosslinking density and the more microporous structure of AC scaffolds enable easier water diffusion into the construct’s interior. Further, increased swelling may lead to increased permeability, resulting in additional water absorption and even faster degradation of the AC scaffold. This leads to significant changes in the mean swelling rates at different time intervals. When immersed in PBS buffer, a gradual exchange of Ca^2+^ with other, monovalent cations (mostly Na^+^) is expected. This effect disrupts the connections between polymer chains and reduces the overall crosslinking density over time. Therefore, it is safe to hypothesize that the greater the starting overall crosslinking density, the lower the scaffolds’ microporosity and, thus, the slower the hydrogel scaffolds’ weight loss. This is also supported by the degradation kinetics of AC and ACC scaffolds alongside the SEM results. For ACC scaffolds, after 30 days, presumably almost all crosslinking connections were broken, which leads to immediate degradation of the scaffold, similar to the behavior observed in the AC scaffolds between 24 and 48 h.

Poor solubility in water and a relatively high melting point are some of the main factors that slow PCL’s degradation rate. Soaking PCL-only scaffolds (P scaffolds) in PBS for up to 32 days had negligible effects on their degradation as they lost only 8% of their initial dry weight. The degradation of PCL, which is a semi-crystalline polyester, involves two stages. Firstly, water diffuses into the amorphous regions, causing a random hydrolytic scission of the ester groups, resulting in additional crystallization and an overall increase in crystallinity. After the amorphous region’s degradation has started, the “hydrolytic attack” shifts towards the crystalline domain center. A molecular weight reduction occurs during water uptake, although it is not accompanied by loss of mass [64]. As therefore expected, the swelling test had negligible effects on PCL due to its hydrophobic properties. The initial increase (approximately 12%) in the scaffold’s weight, which stayed relatively constant for the remainder of the test, can be attributed to either excess PBS, which remained on the scaffold’s surface during weighing, or PBS buffer salts, which precipitated on the P scaffolds.

In contrast, the hybrid ACCP scaffolds lost approximately 20% of their starting weight in the first 48 h, which decreased slightly over the next 29 days. The degradation of the hydrogel component likely contributed the main part of the degradation in the hybrid scaffolds. However, the observed weight loss over time was significantly lower compared to pure hydrogel scaffolds, which can be attributed to two main factors. First, the hybrid scaffolds contain a lower amount of hydrogel. Thus, less material can be degraded. Secondly, the PCL component mechanically stabilizes the scaffold and reduces solvent access to the hydrogel component at the hydrogel–PCL interfaces [64]. Despite a faster degradation rate of ACCP scaffolds than pure P counterparts, mass loss was not critical enough to compromise the scaffold’s overall stability and shape fidelity. The hybrid scaffolds also displayed an intermediate swelling profile. The addition of ALG and CMC into the PCL framework increased the hydrophilic functional groups in the scaffolds, improving water uptake and eventually increasing the swelling ratio. A relatively smaller hydrogel content in hybrid scaffolds than in hydrogel-only scaffolds results in faster swelling and reduced total liquid uptake. Our results suggest that hybrid ACCP scaffolds are especially promising for long-term applications. Furthermore, modulating the ratio of building blocks in the ALG/CMC/PCL hybrid constructs, fine-tuning the hydrogel components’ overall ratios in ACCP scaffolds, and adjusting the post-processing regime may provide a great tool for researchers to tailor scaffolds’ swelling and degradation properties.

#### 3.3.3. Mechanical Properties—Compression and Tensile Tests

**Figure 5:**
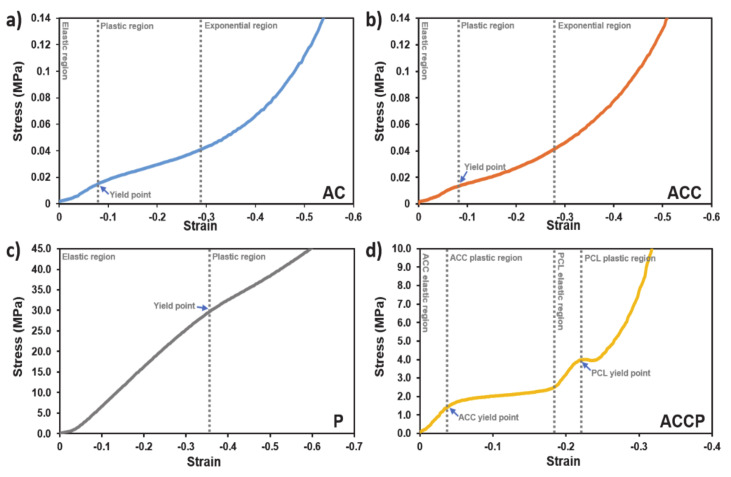
Compressive behavior of the (**a**) AC, (**b**) ACC, (**c**) P, and (**d**) ACCP 3D printed scaffolds. Distinct transition regions of the stress-strain curve are clearly marked for each specimen. The blue arrows indicate the yield points for individual materials.

**Figure 6:**
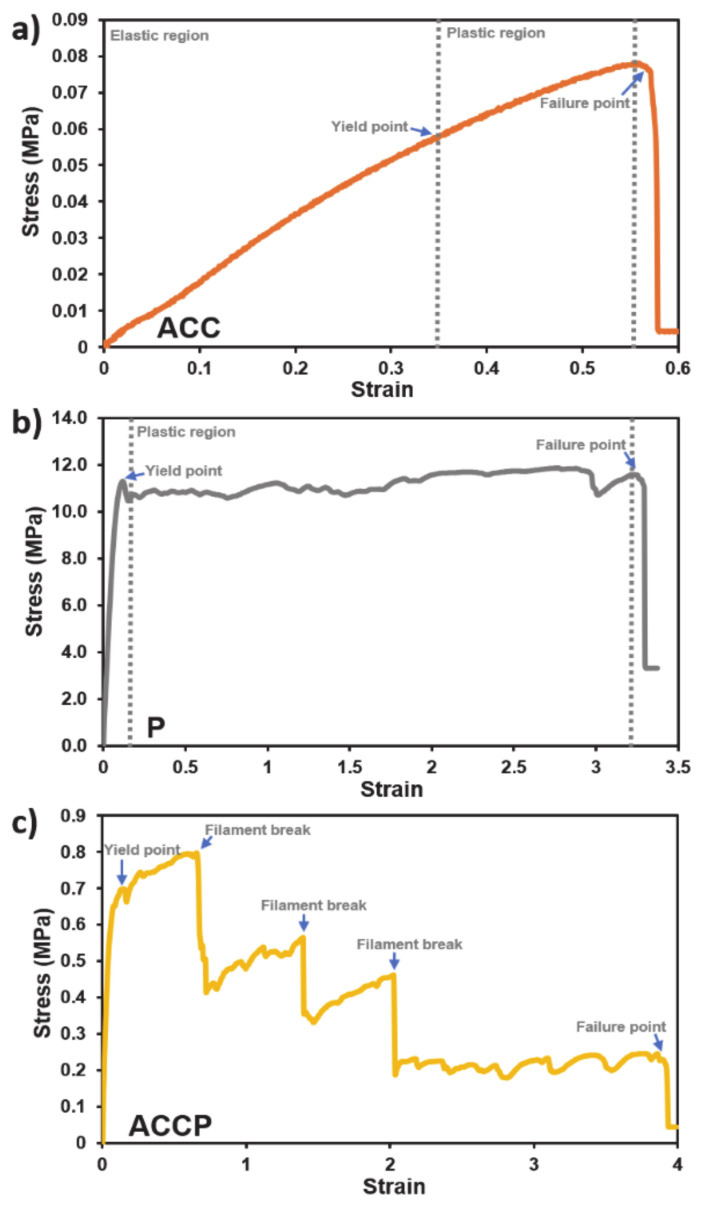
Tensile behavior of analyzed (**a**) ACC, (**b**) P, and (**c**) ACCP 3D printed specimens. Distinct transition regions of the stress-strain curve are clearly marked, whereas the arrows indicate either the yield or failure points, for individual specimens.

In addition to exhibiting appropriate wettability and water uptake capacity, wound dressings must possess suitable mechanical properties. Therefore, the 3D-printed scaffolds were characterized based on their compression and tensile behavior to assess the differences in mechanical properties between base scaffolds. The results of compression and tensile tests have been plotted on stress-strain graphs, which are presented in Figure 5 and Figure 6, respectively, for all scaffolds. In the compression tests, each sample was tested using forces that resulted in the plastic deformation of the specimens in the compression zone. As a result of this, the transition from elastic to plastic deformation could also be observed. All specimens showed a flatter initial response, which can be attributed to the specimens’ surface irregularities resulting from the fabrication process. Only after the compression plate was in contact with the specimen’s full apical surface did the resulting curve start to show the bulk structural response.

Both hydrogel-only scaffolds (AC and ACC) showed similar mechanical behavior in the compression test. The stress-strain curves transitioned at approximately 180 kPa, where the curve flattened slightly (likely due to material breaking and filling the empty spaces in the scaffold). Structural irregularities can thereby cause oscillations within the otherwise smooth curve. Afterward, the curve showed exponential behavior, which is commonly observed in polymers [65]. The transition from the elastic to plastic deformation can only be speculated, since it does not show a characteristic yielding point, as both AC and ACC (regardless of the crosslinking) formulations are extremely soft. Conversely, P scaffolds showed almost linear mechanical behavior in a compression test, which indicates the elastic modulus. A slight plateau can be observed at around −0.35 strain, which represents the yielding point. Afterward, the material started to deform plastically. Hybrid ACCP scaffolds showed more complex compressional mechanical behavior since they combine two already complex materials. The plateau is more evident and corresponds well because the material was fabricated using simultaneous printing of the pure PCL (P) and the AC formulations, and the polymers were printed in each succeeding layer interchangeably. As a result of this, the soft AC was deformed and pushed out of the PCL shell, which is evident in the broad plateau. Afterwards, the PCL shell was deformed, and empty spaces between the printed filament strands started to touch each other and deform elastically again. This is evident in the graph as stiffening. When the yield point of PCL was reached, another plateau can be observed with the PCL component’s successive stiffening. Generally, AC and ACC showed similar mechanical behavior in compression tests, whereas ACCP showed a more complex and especially stiffer behavior compared to the previous specimens. PCL was the stiffest material in comparison to all other specimens.

Tensile tests proved extremely difficult to perform due to the “high hyper-elasticity” of the materials and the low elastic modulus of the materials, since clamping jaws needed to be appropriately adjusted to prevent material slippage in the jaws and avert specimen damage. Nevertheless, after several initial tests, three quality measurements were obtained for each material except AC. The results were again plotted in stress-strain graphs. The tensile behavior of ACC, P, and ACCP scaffolds is presented in Figure 6; however, measurement was rendered impossible for the AC scaffolds.

The results for ACC showed slight local stochastic behavior in the tension region. This behavior can most likely be explained by structural irregularities due to the 3D fabrication process. ACC specimens showed almost linear behavior with low stresses for the given strains. The plastification zone is narrow, which indicates that the material deformed elastically and then broke suddenly, with little plastification. On the contrary, PCL was much stiffer compared to ACC and showed a higher elastic modulus. The yield point is around 11 MPa, and afterward a broad plastification zone begins. The plastification occurs at almost the same stress, which indicates that the material was stiffening due to the mechanisms occurring during plastic deformation. The strain at the material’s failure point is above 3, which is five times higher than that of ACC. ACCP again showed a combination of both the AC and PCL component. The curve’s initial slope, indicating the PCL component’s elastic modulus, is similar to the PCL-only scaffold. The lower yield strength of the ACCP material at ~0.7 MPa can be attributed to the fact that the specimen was manufactured as a combination of PCL and ACC. Since the test specimen’s size was the same as that of the specimen of homogeneous PCL, the hybrid ACCP showsed a smaller cross-sectional area of PCL than that of solid PCL. Therefore, the PCL’s true stress was much higher, probably comparable to the PCL specimen test. The yield point was clearly visible with the visible strengthening of the specimen. After critical stress was reached, a filament strand inside the specimen broke. The specimen settled at a lower stress value, where the strengthening of the specimen with increasing strain began again. This effect can be attributed to the structure of the PCL strands, which were not interconnected. The behavior was repeated until all filaments of the scaffold broke. The strain value at which the specimen failed was much higher than that observed in ACC and was similar to that of the PCL specimen. Generally, PCL was the stiffest material, with a broad plastification zone, and ACCP was effectively softer for the given area and showed a characteristic plastification zone. ACC showed the lowest elastic modulus, with the lowest yield point and sudden breakage.

Based on the compression and tensile experiments, it is evident that ionic crosslinking influences the mechanical behavior of scaffolds (although only slightly). More importantly, scaffolds’ stiffness and durability to mechanical forces are significantly improved when the soft hydrogel is incorporated into a solid PCL framework. This suggests that mechanical characteristics of wound dressings can be tailored merely by adjusting the mass ratios of the hydrogel and synthetic components in the scaffolds. Our results indicate that hybrid 3D printing may present a great approach for researchers to regulate and fine-tune the scaffold’s mechanical characteristics to mimic native tissues’ characteristics or develop more durable wound dressings.

#### 3.3.4. Viability of Skin Cells

**Figure 7:**
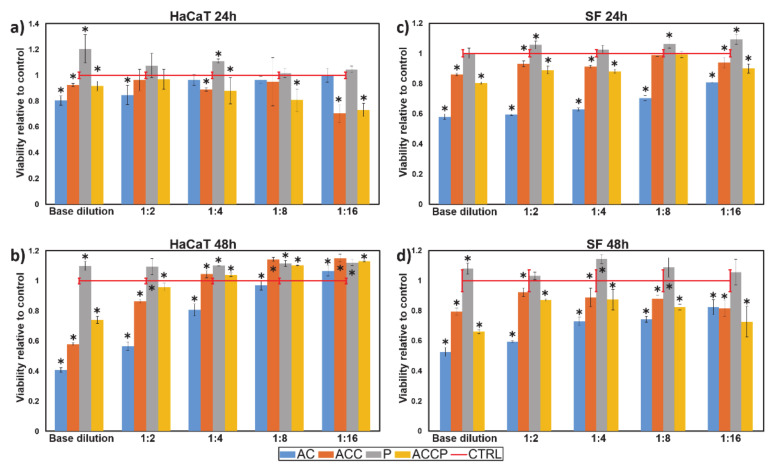
Results of the MTT biocompatibility assay for keratinocytes (HaCaTs) (**a**,**b**) and human skin-derived fibroblasts (SFs) (**c**,**d**) obtained after 24-h and 48-h incubation periods. The asterisks indicate where the differences between the sample and the control were statistically significant (* p < 0.05).

The skin is a continuously self-renewing organ that dynamically copes with the human body’s exterior–interior interactions and actively participates in the host defenses [6]. Ideally, wound dressings should provide a protective and regenerative function to promote cell growth and healing with no negative effects on the underlying tissue. Therefore, a crucial aspect of the characterization was the viability testing of skin cells (by assessing their cellular metabolic activity) when in contact with the scaffolds, evaluated using the MTT assay. For this purpose, human skin-derived fibroblasts (SF) and keratinocytes (HaCaT) were tested in contact with all formulations. The combined results of the MTT assay for both cell lines (HaCaTs and SFs) at two discrete time points (24 h and 48 h) are presented in Figure 7.

As the MTT assay reflects the total metabolic activity of cells in a sample, variances in the initial cell concentrations per sample are to be expected, resulting in fluctuations between samples. Extrapolating from the obtained data thus requires very careful consideration. Due to their poor solubility, extracts from P scaffolds showed little effect on cell viability. However, despite showcasing great biocompatibility, PCL’s hydrophobic nature impeded cell attachment. Although providing a stable environment for cell growth, a pure PCL scaffold is therefore not the best material for fabricating wound dressings [3]. All other scaffolds significantly affected the MTT results, which can be attributed to their chemistry. Although the scaffold extracts showed a small effect on the HaCaT cells after 24 h, cell viability decreased with increasing concentrations, especially for AC scaffolds. Considering the degradation kinetics, these scaffolds’ extracts were also likely to contain the highest amount of dissolved polymers and to release the highest amount of Ca^2+^. Similarly, extracts from AC scaffolds showed the largest impact on SF viability, reducing metabolic activity with increasing concentration. However, the results after 24 and 48 h remained comparable.

Degradation is most likely the consequence of cation exchange in growth media (Na^+^ from growth media for Ca^2+^ from the scaffold). The outcome of this cation exchange is a complete dissolution of ALG and CMC, which leads to extensive material transformation and eventually to its degradation [3]. Initially, we were surprised by the poor performance of the AC and ACC scaffold formulations, as both ALG and CMC were previously proven to be biocompatible [62]. To explain the unanticipated results, we prepared an ALG-CMC solution (1 mg/mL) and measured its respective pH value to be 6.6, which explains and is in perfect agreement with our MTT results. Lower-than-physiological pH values are unfavorable for cell viability [62]. Therefore, both cell types’ considerable negative effects can be attributed to the medium’s lower pH values due to the dissolution of ALG and CMC. Both ALG and CMC are water-soluble “poly-carboxylic acids”, yet the main drop in medium’s pH can be ascribed to ALG. ALG has a higher ratio of carboxyl groups per anhydro-glucose unit because of its smaller monomer molecular weight (C_6_H_7_O_6_Na; Mw: 198.0 g/mol), in comparison with a single CMC monomer unit (C_8_H_15_O_8_Na; Mw: 262.2 g/mol). CMC has 0.9 carboxyl groups per one anhydro-glucose unit. Additionally, a single CMC polymer chain’s molecular weight is approximately nine times larger (700 kDa vs. 80 kDa) than a single ALG polymer chain, which probably also influences its dissolution [62]. The inequality in the carboxyl group ratio per one monomer unit is, therefore, further emphasized. The greater influence of ALG on the medium’s pH was experimentally confirmed by measuring the pH values of pure ALG and CMC solutions (both 1 mg/mL); the ALG solution’s pH value was measured to be 6.3 and 7.0 for CMC. Therefore, the drop in pH can be mitigated by fine-tuning the ALG-CMC weight ratio in scaffolds favoring CMC, so that the pH reduction is not as severe. Taking this into consideration, it is expected that both SFs and HaCaTs exposed to AC scaffolds showed the lowest viability.

Therefore, it was also anticipated (and confirmed by MTT results) that the additionally crosslinked ACC scaffolds, which degrade significantly slower than their non-post-processed counterparts (AC scaffolds), influence cell viability significantly less. The ACC scaffolds lost approximately 40% of their weight in the first 48 h. Thus, it can be hypothesized that much lower amounts of ALG and CMC are released into the growth medium. Nevertheless, because the ACC scaffolds’ degradation is not as extensive and the medium’s pH values are not lowered as considerably, the significantly lower viability of HaCaTs when exposed to ACC scaffolds can be attributed to another key factor. Namely, lower viability can be partially related to the excess Ca^2+^ in the growth medium (as a consequence of cation exchange) of ACC extracts instead of lower pH values due to scaffold degradation. Previous studies showed that cultured HaCaT cells can switch between differentiated and basal states upon alterations in the Ca^2+^ concentration in media. The addition of Ca^2+^ triggers HaCaT hyperproliferation. When exposed to extracellular Ca^2+^, HaCaTs most likely undergo a spontaneous transformation and become relatively insensitive to Ca^2+^, which normally triggers reduced proliferation through the differentiation step [66]. Degradation of ACC scaffolds produces a high Ca^2+^ environment, which induces the proliferation of HaCaT cells, which, after the formation of a cell monolayer, is decreased in favor of rapid differentiation. Once their differentiation is initiated, HaCaTs begin to slowly undergo apoptosis [67], which is first showcased as a significant decline in cell viability (also observed in the MTT results). This observation further supports the statement that cell viability seems to increase with increasing extract dilutions. A closer look at the 48-h MTT results indicated that SFs may be more susceptible to changes in the medium’s pH values and alterations in Ca^2+^ concentration, as their viability did not improve with increasing extract dilutions, regardless of the scaffold’s composition.

If we exclude the P scaffolds, the most biocompatible scaffold seems to be the hybrid ACCP scaffold. In the first 24 h of incubation, no significant differences for either cell line between the ACC and the ACCP scaffolds were observed; however, after 48 h, significantly higher cell viabilities (for both exposed cell types, HaCaTs and SFs) were noticed for ACCP scaffolds. This result is also in agreement with the degradation results. For hybrid ACCP scaffolds, the viability was not decreased during the first 24 h, as only a small amount of the additionally crosslinked ALG-CMC component degraded. The total amount of the degraded ALG and CMC was too small to change the medium’s pH to the degree that would influence SF and HaCaT cells’ viability. The slower degradation process of the crosslinked hydrogel component, and lower ALG-CMC content per scaffold than hydrogel-only scaffolds, means that the pH of the growth media was maintained closer to physiological values. This also dictates a lower Ca^2+^ concentration. This resulted in significantly higher HaCaT viability after 48 h, relative to ACC scaffolds. Cell viability further increased with larger extract dilutions and even surpasses the control cells. The hybrid ACCP results again support the statement that SF cells, on the other hand, seem to be much more sensitive to alterations in pH values or Ca^2+^ concentrations, as no increasing trend was observed.

All things considered, it appears that the scaffold’s composition and degradation properties, combined with the cell type’s susceptibility to environmental changes (pH, the concentration of different ions), greatly affect cell viability outcomes. Therefore, when designing potential scaffolds for wound dressings or skin substitutes, especially in the design of infrequently changed dressings, one must first consider the cell type whose growth the scaffold is supposed to promote. The scaffold’s formulation and fabrication should be fine-tuned accordingly. In the case of skin-derived cells, even though the P scaffold’s biocompatibility is the highest, due to PCL’s inherently unfavorable properties, the best scaffold choice for supporting their growth is the hybrid ACCP scaffold. This form of scaffold combines the highly biocompatible PCL, which is crucial in long-term cell culturing (as it retains its integrity), with hydrogels’ desirable physicochemical properties. AC scaffolds show that small concentrations of hydrogel base components (ALG and CMC) improve long-term cell viability. As evident from our evaluation of AC and ACC scaffolds, additional ionic crosslinking of the hydrogel component improves hydrogels’ stability in the culture medium. ACC and ACCP scaffolds serve as an indication that appropriate post-processing of scaffolds delays hydrogel dissolution and mitigates pH changes. However, when ionically crosslinking hydrogels, we must also consider the subsequent release of ions (such as Ca^2+^) into the growth medium. As seen in the results from different cell types, the crosslinking solution should be tailored according to the materials used and fine-tuned based on the cell types that are in contact with the scaffolds. Finally, it is important to stress that all the prepared formulations displayed no visible cytotoxic effects on cells. Considering all results, the hybrid ACCP formulation demonstrated desirable properties and appropriate in vitro biocompatibility, making it the best candidate for wound healing applications.

## 4. Conclusions

In this work, a hybrid extrusion-based 3D printing approach was employed to fabricate scaffolds that could serve as advanced wound dressings with tunable fundamental properties. Firstly, a hydrogel formulation based on two of the most commonly used polysaccharides in wound care (alginate and carboxymethyl cellulose) was prepared and optimized for 3D printing. The optimized hydrogel served as a “wound dressing template”, which was integrated into the synthetic polycaprolactone framework.

By optimizing the 3D printing procedure and crosslinking, hybrid scaffolds with improved mechanical and physicochemical properties were fabricated. The hydrogel formulation was simultaneously printed with polycaprolactone in an alternating sequence. Systematic material and scaffold characterization tests were conducted to examine the influence of ionic-crosslinking and integration of the hydrogel component in the stiff synthetic framework. The methods used included evaluating the resulting material’s structural properties and the scaffold’s stability, durability, and mechanical behavior. Using a state-of-the-art printing strategy and employing a multi-head deposition system, we proved that combining soft hydrogels with stiff polymers into a single construct presents a straightforward approach to producing macroporous, highly organized, mechanically superior hybrid scaffolds. We further demonstrated that fabrication by sequential dispensing of the thermoplastic PCL and the ALG/CMC hydrogel fulfills both biomaterials’ thermal requirements and facilitates integrating polymers with different requirements into a single construct. Compared to hydrogel-only scaffolds, the hybrid constructs displayed significantly improved mechanical properties (improved from 0.2 MPa to 11 MPa), degradation kinetics (improved from approx. 2 days to 30+ days), appropriate water uptake capacity, wettability, and good surface morphology characteristics. Additionally, by carefully optimizing and adjusting the printing parameters, the post-processing regime, and ratios of both the hydrogel and synthetic components in the constructs, the hybrid approach enables rather simple control over the scaffolds’ characteristics. The mentioned parameters can be adjusted to fine-tune them for specific applications. The prepared 3D printed wound dressings were proven to be safe for use by measuring skin-derived cells’ viability when exposed to scaffolds with the MTT assay. All scaffolds were proven to be biocompatible and exerted no toxic effects on skin-derived cells. Based on the results, this study lays the groundwork for developing biocompatible hybrid scaffolds that can serve as advanced wound dressings with improved properties. Moreover, the hybrid printing approach used in this study opens up the possibility of additionally tuning the properties of scaffolds, so that they can more closely mimic the cells’ native microenvironment, thus guiding the cell behavior to promote the wound-healing process.

## Figures and Tables

**Table 1:** Measured filament thickness, pore dimensions, and calculated macroporosity of the 3D printed scaffolds.

Parameters	AC	ACC	P	ACCP
Average filament thickness (mm)	0.49 ± 0.04	0.38 ± 0.07	0.28 ± 0.08	0.33 ± 0.16
Average pore width (mm)	0.09 ± 0.07	0.19 ± 0.07	0.95 ± 0.02	0.17 ± 0.03
Average pore length (mm)	0.09 ± 0.04	0.20 ± 0.06	0.93 ± 0.01	0.50 ± 0.02
Average pore area (mm^2^)	0.007 ± 0.007	0.024 ± 0.012	0.768 ± 0.033	0.077 ± 0.014
Macroporosity (%)	1.53	9.73	56.78	15.72

**Table 2:** Average water contact angle (CA) values with corresponding standard deviations for respective formulations.

AC	ACC	P	ACCP
CA = 50.0 ± 3.8° 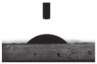	CA = 13.7 ± 1.6° 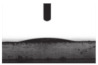	CA = 75.9 ± 0.9° 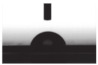	CA = 66.9 ± 2.2° 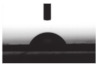

## Data Availability

The data presented in this study are available within the article.
